# Supplementary material for: HIVprotI: an integrated web based platform for prediction and design of HIV proteins inhibitors
Source: J Cheminform. 2018 Mar 9;10:12. doi: 10.1186/s13321-018-0266-y (PMC5845081; doi:10.1186/s13321-018-0266-y)
Supplement: Supplementary file 2 — Additional file 2. Source code of HIVProtI web server. [file 13321_2018_266_MOESM2_ESM.zip › HIVprotI_Source-code/hivprotI/weka-3-6-6/doc/weka/filters/unsupervised/attribute/NumericCleaner.html]

NumericCleaner


---


|  |  |  |
| --- | --- | --- |
| **Overview** \| **Package** \| **Class** \| **Tree** \| **Deprecated** \| **Index** \| **Help** \| **Weka's home** | |  |
| **PREV CLASS**   **NEXT CLASS** | **FRAMES**    **NO FRAMES**     **All Classes** |  |
| SUMMARY: NESTED \| FIELD \| CONSTR \| METHOD | DETAIL: FIELD \| CONSTR \| METHOD |  |


---


## weka.filters.unsupervised.attribute Class NumericCleaner

```
java.lang.Object
  weka.filters.Filter
      weka.filters.SimpleFilter
          weka.filters.SimpleStreamFilter
              weka.filters.unsupervised.attribute.NumericCleaner
```

**All Implemented Interfaces:**: java.io.Serializable, CapabilitiesHandler, OptionHandler, RevisionHandler, StreamableFilter

---

``` public class NumericCleaner extends SimpleStreamFilter ```

A filter that 'cleanses' the numeric data from values that are too small, too big or very close to a certain value (e.g., 0) and sets these values to a pre-defined default.


Valid options are:

```
 -D
  Turns on output of debugging information.
```

```
 -min <double>
  The minimum threshold. (default -Double.MAX_VALUE)
```

```
 -min-default <double>
  The replacement for values smaller than the minimum threshold.
  (default -Double.MAX_VALUE)
```

```
 -max <double>
  The maximum threshold. (default Double.MAX_VALUE)
```

```
 -max-default <double>
  The replacement for values larger than the maximum threshold.
  (default Double.MAX_VALUE)
```

```
 -closeto <double>
  The number values are checked for closeness. (default 0)
```

```
 -closeto-default <double>
  The replacement for values that are close to '-closeto'.
  (default 0)
```

```
 -closeto-tolerance <double>
  The tolerance below which numbers are considered being close to 
  to each other. (default 1E-6)
```

```
 -decimals <int>
  The number of decimals to round to, -1 means no rounding at all.
  (default -1)
```

```
 -R <col1,col2,...>
  The list of columns to cleanse, e.g., first-last or first-3,5-last.
  (default first-last)
```

```
 -V
  Inverts the matching sense.
```

```
 -include-class
  Whether to include the class in the cleansing.
  The class column will always be skipped, if this flag is not
  present. (default no)
```

**Version:**
:   $Revision: 5543 $

**Author:**
:   fracpete (fracpete at waikato dot ac dot nz)

**See Also:**: Serialized Form

---

**Constructor Summary**

`NumericCleaner()`


| **Method Summary** | |
| --- | --- |
| `java.lang.String` | `attributeIndicesTipText()`             Returns the tip text for this property |
| `java.lang.String` | `closeToDefaultTipText()`             Returns the tip text for this property |
| `java.lang.String` | `closeToTipText()`             Returns the tip text for this property |
| `java.lang.String` | `closeToToleranceTipText()`             Returns the tip text for this property |
| `java.lang.String` | `decimalsTipText()`             Returns the tip text for this property |
| `java.lang.String` | `getAttributeIndices()`             Gets the selection of the columns, e.g., first-last or first-3,5-last |
| `Capabilities` | `getCapabilities()`             Returns the Capabilities of this filter. |
| `double` | `getCloseTo()`             Get the "close to" number. |
| `double` | `getCloseToDefault()`             Get the "close to" default. |
| `double` | `getCloseToTolerance()`             Get the "close to" Tolerance. |
| `int` | `getDecimals()`             Get the number of decimals to round to. |
| `boolean` | `getIncludeClass()`             Gets whether the class is included in the cleaning process or always skipped. |
| `boolean` | `getInvertSelection()`             Gets whether the selection of the columns is inverted |
| `double` | `getMaxDefault()`             Get the maximum default. |
| `double` | `getMaxThreshold()`             Get the maximum threshold. |
| `double` | `getMinDefault()`             Get the minimum default. |
| `double` | `getMinThreshold()`             Get the minimum threshold. |
| `java.lang.String[]` | `getOptions()`             Gets the current settings of the filter. |
| `java.lang.String` | `getRevision()`             Returns the revision string. |
| `java.lang.String` | `globalInfo()`             Returns a string describing this filter. |
| `java.lang.String` | `includeClassTipText()`             Returns the tip text for this property |
| `java.lang.String` | `invertSelectionTipText()`             Returns the tip text for this property |
| `java.util.Enumeration` | `listOptions()`             Returns an enumeration describing the available options. |
| `static void` | `main(java.lang.String[] args)`             Runs the filter from commandline, use "-h" to see all options. |
| `java.lang.String` | `maxDefaultTipText()`             Returns the tip text for this property |
| `java.lang.String` | `maxThresholdTipText()`             Returns the tip text for this property |
| `java.lang.String` | `minDefaultTipText()`             Returns the tip text for this property |
| `java.lang.String` | `minThresholdTipText()`             Returns the tip text for this property |
| `void` | `setAttributeIndices(java.lang.String value)`             Sets the columns to use, e.g., first-last or first-3,5-last |
| `void` | `setCloseTo(double value)`             Set the "close to" number. |
| `void` | `setCloseToDefault(double value)`             Set the "close to" default. |
| `void` | `setCloseToTolerance(double value)`             Set the "close to" Tolerance. |
| `void` | `setDecimals(int value)`             Set the number of decimals to round to. |
| `void` | `setIncludeClass(boolean value)`             Sets whether the class can be cleaned, too. |
| `void` | `setInvertSelection(boolean value)`             Sets whether the selection of the indices is inverted or not |
| `void` | `setMaxDefault(double value)`             Set the naximum default. |
| `void` | `setMaxThreshold(double value)`             Set the maximum threshold. |
| `void` | `setMinDefault(double value)`             Set the minimum default. |
| `void` | `setMinThreshold(double value)`             Set the minimum threshold. |
| `void` | `setOptions(java.lang.String[] options)`             Parses a given list of options. |

**Methods inherited from class weka.filters.SimpleStreamFilter**

`batchFinished, input`

**Methods inherited from class weka.filters.SimpleFilter**

`debugTipText, getDebug, setDebug, setInputFormat`

**Methods inherited from class weka.filters.Filter**

`batchFilterFile, filterFile, getCapabilities, getOutputFormat, isFirstBatchDone, isNewBatch, isOutputFormatDefined, makeCopies, makeCopy, numPendingOutput, output, outputPeek, toString, useFilter, wekaStaticWrapper`

**Methods inherited from class java.lang.Object**

`equals, getClass, hashCode, notify, notifyAll, wait, wait, wait`

**Constructor Detail**

### NumericCleaner

```
public NumericCleaner()
```


**Method Detail**

### globalInfo

```
public java.lang.String globalInfo()
```

:   Returns a string describing this filter.

    :   **Specified by:**: `globalInfo` in class `SimpleFilter`
    :   **Returns:**: a description of the filter suitable for displaying in the explorer/experimenter gui

---


### listOptions

```
public java.util.Enumeration listOptions()
```

:   Returns an enumeration describing the available options.

    :   **Specified by:**: `listOptions` in interface `OptionHandler` **Overrides:**: `listOptions` in class `SimpleFilter`
    :   **Returns:**: an enumeration of all the available options.

---


### getOptions

```
public java.lang.String[] getOptions()
```

:   Gets the current settings of the filter.

    :   **Specified by:**: `getOptions` in interface `OptionHandler` **Overrides:**: `getOptions` in class `SimpleFilter`
    :   **Returns:**: an array of strings suitable for passing to setOptions

---


### setOptions

```
public void setOptions(java.lang.String[] options)
                throws java.lang.Exception
```

:   Parses a given list of options.


    Valid options are:

    ```
     -D
      Turns on output of debugging information.
    ```

    ```
     -min <double>
      The minimum threshold. (default -Double.MAX_VALUE)
    ```

    ```
     -min-default <double>
      The replacement for values smaller than the minimum threshold.
      (default -Double.MAX_VALUE)
    ```

    ```
     -max <double>
      The maximum threshold. (default Double.MAX_VALUE)
    ```

    ```
     -max-default <double>
      The replacement for values larger than the maximum threshold.
      (default Double.MAX_VALUE)
    ```

    ```
     -closeto <double>
      The number values are checked for closeness. (default 0)
    ```

    ```
     -closeto-default <double>
      The replacement for values that are close to '-closeto'.
      (default 0)
    ```

    ```
     -closeto-tolerance <double>
      The tolerance below which numbers are considered being close to 
      to each other. (default 1E-6)
    ```

    ```
     -decimals <int>
      The number of decimals to round to, -1 means no rounding at all.
      (default -1)
    ```

    ```
     -R <col1,col2,...>
      The list of columns to cleanse, e.g., first-last or first-3,5-last.
      (default first-last)
    ```

    ```
     -V
      Inverts the matching sense.
    ```

    ```
     -include-class
      Whether to include the class in the cleansing.
      The class column will always be skipped, if this flag is not
      present. (default no)
    ```

    :   **Specified by:**: `setOptions` in interface `OptionHandler` **Overrides:**: `setOptions` in class `SimpleFilter`
    :   **Parameters:**: `options` - the list of options as an array of strings **Throws:**: `java.lang.Exception` - if an option is not supported **See Also:**: `SimpleFilter.reset()`

---


### getCapabilities

```
public Capabilities getCapabilities()
```

:   Returns the Capabilities of this filter.

    :   **Specified by:**: `getCapabilities` in interface `CapabilitiesHandler` **Overrides:**: `getCapabilities` in class `Filter`
    :   **Returns:**: the capabilities of this object **See Also:**: `Capabilities`

---


### minThresholdTipText

```
public java.lang.String minThresholdTipText()
```

:   Returns the tip text for this property

    :   **Returns:**: tip text for this property suitable for displaying in the explorer/experimenter gui

---


### getMinThreshold

```
public double getMinThreshold()
```

:   Get the minimum threshold.

    :   **Returns:**: the minimum threshold.

---


### setMinThreshold

```
public void setMinThreshold(double value)
```

:   Set the minimum threshold.

    :   **Parameters:**: `value` - the minimum threshold to use.

---


### minDefaultTipText

```
public java.lang.String minDefaultTipText()
```

:   Returns the tip text for this property

    :   **Returns:**: tip text for this property suitable for displaying in the explorer/experimenter gui

---


### getMinDefault

```
public double getMinDefault()
```

:   Get the minimum default.

    :   **Returns:**: the minimum default.

---


### setMinDefault

```
public void setMinDefault(double value)
```

:   Set the minimum default.

    :   **Parameters:**: `value` - the minimum default to use.

---


### maxThresholdTipText

```
public java.lang.String maxThresholdTipText()
```

:   Returns the tip text for this property

    :   **Returns:**: tip text for this property suitable for displaying in the explorer/experimenter gui

---


### getMaxThreshold

```
public double getMaxThreshold()
```

:   Get the maximum threshold.

    :   **Returns:**: the maximum threshold.

---


### setMaxThreshold

```
public void setMaxThreshold(double value)
```

:   Set the maximum threshold.

    :   **Parameters:**: `value` - the maximum threshold to use.

---


### maxDefaultTipText

```
public java.lang.String maxDefaultTipText()
```

:   Returns the tip text for this property

    :   **Returns:**: tip text for this property suitable for displaying in the explorer/experimenter gui

---


### getMaxDefault

```
public double getMaxDefault()
```

:   Get the maximum default.

    :   **Returns:**: the maximum default.

---


### setMaxDefault

```
public void setMaxDefault(double value)
```

:   Set the naximum default.

    :   **Parameters:**: `value` - the maximum default to use.

---


### closeToTipText

```
public java.lang.String closeToTipText()
```

:   Returns the tip text for this property

    :   **Returns:**: tip text for this property suitable for displaying in the explorer/experimenter gui

---


### getCloseTo

```
public double getCloseTo()
```

:   Get the "close to" number.

    :   **Returns:**: the "close to" number.

---


### setCloseTo

```
public void setCloseTo(double value)
```

:   Set the "close to" number.

    :   **Parameters:**: `value` - the number to use for checking closeness.

---


### closeToDefaultTipText

```
public java.lang.String closeToDefaultTipText()
```

:   Returns the tip text for this property

    :   **Returns:**: tip text for this property suitable for displaying in the explorer/experimenter gui

---


### getCloseToDefault

```
public double getCloseToDefault()
```

:   Get the "close to" default.

    :   **Returns:**: the "close to" default.

---


### setCloseToDefault

```
public void setCloseToDefault(double value)
```

:   Set the "close to" default.

    :   **Parameters:**: `value` - the "close to" default to use.

---


### closeToToleranceTipText

```
public java.lang.String closeToToleranceTipText()
```

:   Returns the tip text for this property

    :   **Returns:**: tip text for this property suitable for displaying in the explorer/experimenter gui

---


### getCloseToTolerance

```
public double getCloseToTolerance()
```

:   Get the "close to" Tolerance.

    :   **Returns:**: the "close to" Tolerance.

---


### setCloseToTolerance

```
public void setCloseToTolerance(double value)
```

:   Set the "close to" Tolerance.

    :   **Parameters:**: `value` - the "close to" Tolerance to use.

---


### attributeIndicesTipText

```
public java.lang.String attributeIndicesTipText()
```

:   Returns the tip text for this property

    :   **Returns:**: tip text for this property suitable for displaying in the explorer/experimenter gui

---


### getAttributeIndices

```
public java.lang.String getAttributeIndices()
```

:   Gets the selection of the columns, e.g., first-last or first-3,5-last

    :   **Returns:**: the selected indices

---


### setAttributeIndices

```
public void setAttributeIndices(java.lang.String value)
```

:   Sets the columns to use, e.g., first-last or first-3,5-last

    :   **Parameters:**: `value` - the columns to use

---


### invertSelectionTipText

```
public java.lang.String invertSelectionTipText()
```

:   Returns the tip text for this property

    :   **Returns:**: tip text for this property suitable for displaying in the explorer/experimenter gui

---


### getInvertSelection

```
public boolean getInvertSelection()
```

:   Gets whether the selection of the columns is inverted

    :   **Returns:**: true if the selection is inverted

---


### setInvertSelection

```
public void setInvertSelection(boolean value)
```

:   Sets whether the selection of the indices is inverted or not

    :   **Parameters:**: `value` - the new invert setting

---


### includeClassTipText

```
public java.lang.String includeClassTipText()
```

:   Returns the tip text for this property

    :   **Returns:**: tip text for this property suitable for displaying in the explorer/experimenter gui

---


### getIncludeClass

```
public boolean getIncludeClass()
```

:   Gets whether the class is included in the cleaning process or always
    skipped.

    :   **Returns:**: true if the class can be considered for cleaning.

---


### setIncludeClass

```
public void setIncludeClass(boolean value)
```

:   Sets whether the class can be cleaned, too.

    :   **Parameters:**: `value` - true if the class can be cleansed, too

---


### decimalsTipText

```
public java.lang.String decimalsTipText()
```

:   Returns the tip text for this property

    :   **Returns:**: tip text for this property suitable for displaying in the explorer/experimenter gui

---


### getDecimals

```
public int getDecimals()
```

:   Get the number of decimals to round to.

    :   **Returns:**: the number of decimals.

---


### setDecimals

```
public void setDecimals(int value)
```

:   Set the number of decimals to round to.

    :   **Parameters:**: `value` - the number of decimals.

---


### getRevision

```
public java.lang.String getRevision()
```

:   Returns the revision string.

    :   **Specified by:**: `getRevision` in interface `RevisionHandler` **Overrides:**: `getRevision` in class `Filter`
    :   **Returns:**: the revision

---


### main

```
public static void main(java.lang.String[] args)
```

:   Runs the filter from commandline, use "-h" to see all options.

    :   **Parameters:**: `args` - the commandline options for the filter


---


|  |  |  |
| --- | --- | --- |
| **Overview** \| **Package** \| **Class** \| **Tree** \| **Deprecated** \| **Index** \| **Help** \| **Weka's home** | |  |
| **PREV CLASS**   **NEXT CLASS** | **FRAMES**    **NO FRAMES**     **All Classes** |  |
| SUMMARY: NESTED \| FIELD \| CONSTR \| METHOD | DETAIL: FIELD \| CONSTR \| METHOD |  |


---
